# Pediatric Health Risk Assessment for Exposure to Aluminum from Infant Formulas and Children under the Age of Five’s Food Products among Arab Infants: Experience from Lebanon

**DOI:** 10.3390/foods11162503

**Published:** 2022-08-19

**Authors:** Carla Ibrahim, Zeinab Kammouni, Maryam Barake, Mounir Kassir, Ayoub Al-Jawaldeh, Joseph Matta, Yonna Sacre, Lara Hanna-Wakim, Joyce Haddad, Maha Hoteit

**Affiliations:** 1 Doctoral School of Sciences and Technology (DSST), Lebanese University, Hadath 6573, Lebanon; 2 Faculty of Public Health, Section 1, Lebanese University, Beirut 6573, Lebanon; 3 PHENOL Research Group (Public HEalth Nutrition prOgram Lebanon), Faculty of Public Health, Lebanese University, Beirut 6573, Lebanon; 4 Lebanese University Nutrition Surveillance Center (LUNSC), Lebanese Food Drugs and Chemical Administrations, Lebanese University, Beirut 6573, Lebanon; 5 Department of Nutrition and Food Sciences, Faculty of Arts and Sciences, Holy Spirit University of Kaslik (USEK), Jounieh P.O. Box 446, Lebanon; 6 Platform for Research and Analysis in Environmental Sciences, Doctoral School of Science and Technology (DSST), Lebanese University, Beirut 6573, Lebanon; 7 World Health Organization Regional Office for the Eastern Mediterranean, Cairo 11371, Egypt; 8 Department of Nutrition, Faculty of Pharmacy, Saint-Joseph University of Beirut, Medical Sciences Campus, Damascus Road, P.O. Box 11-5076, Riad Solh, Beirut 1107 2180, Lebanon; 9 Industrial Research Institute, Lebanese University Campus, Hadath 6573, Lebanon; 10 Department of Agricultural and Food Engineering, School of Engineering, Holy Spirit University of Kaslik (USEK), Jounieh P.O. Box 446, Lebanon; 11 Directorate of Preventive Healthcare, Ministry of Public Health, Beirut 6573, Lebanon

**Keywords:** aluminum, occurrence, exposure, health risk assessment, infant formula, complementary food, Lebanon

## Abstract

Chronic dietary aluminum (Al) exposure can have various negative effects on health. The aim of our study is to (1) assess the contamination level of Al in infant formulas (*n* = 41) and baby food products (*n* = 76) available in the Lebanese market, and to (2) evaluate the margin of exposure of Al through the consumption of these foods among children under the age of five in Lebanon. Flame atomic absorption spectrometry (FAAS) was used to evaluate all of the samples. Al levels in all tested children’s food items were below the limit of detection. The highest Al level was detected in cornflakes (0.361 ± 0.049 mg/kg) and pureed foods (0.362 ± 0.079 mg/kg). Among infants aged 0–23 months, the average Al exposure due to the daily intake of infant formulas and baby foods was 0.01 and 0.0104 mg/kg BW/day for males and females, respectively. Babies aged 8–10 and 3–5 months had the highest and lowest levels of Al exposure, respectively. Additionally, the toxicological contribution of Al exposure determined for several age groups to a provisional tolerated weekly intake (PTWI) set by JECFA was  <6% and  <7% for males and females, respectively. The total Al exposure through the consumption of infant formulas and complementary foods among all ages in both males and females was below the values of weekly tolerable intakes (2 mg/kg/BW/W) set by JECFA. However, the values of hazard quotient (HQ) exceeded 1 in both male and female Lebanese infants. As a result, the risk of infants being exposed to Al in baby foods needs to be continuously considered.

## 1. Introduction

Breastfeeding is the optimal food for infants and young children. It provides many health advantages, lowers disease risk, and improves the infant’s nutritional status [1]. The most well-known milk substitutes are infant formulas that are used to replace breastmilk to cover an infant’s nutritional requirements during the first few months of life until the appropriate introduction of complementary feeding [2]. However, infant formulas may contain pollutants, such as heavy metals, that pose a health risk to infants and young children through their bioaccumulation mechanism [3]. Cow’s milk, which comprises the majority of infant formulas, may include these hazardous metals as a result of the cow’s consumption of tainted feedstuffs and water, and exposure to pollution. Further, water, utensils, containers, and equipment used in the manufacture, packing, and storage of infant formulas and complementary foods (such as pureed fruits, vegetables, and meat, and infantile cereals and biscuits) are additional sources of contaminants [4]. Because of their unique physiology, toxicokinetics, body weight (BW) ratio, and rapid growth, infants are more vulnerable to hazardous chemical pollutants such as aluminum (Al) [2].

Al is a common element in the hard layer of Earth that has long been thought to be harmless and safe for humans due to its limited bioavailability [5]. However, epidemiological data linking chronic Al exposure to Alzheimer’s disease cast doubt on its safety [6]. Additionally, chronic dietary Al consumption can have negative neurologic, skeletal, hematopoietic, immunologic, and other health impacts [6]. The Joint Food and Agricultural Organization/World Health Organization (WHO) Expert Committee on Food Additives (JECFA) set a provisional tolerated weekly intake (PTWI) of Al of 2 mg/kg BW/week [7]. Al comprises 8.8% (88 g/kg) of the hard layer of Earth by mass, and it can be found in a variety of rocks; it is usually released into the environment as a result of the natural weathering of rocks [8]. Since Al can be dissolved in acidic conditions, acid rain can lead to toxic levels of dissolved Al in the surrounding water and soils [8,9]. Due to its high reactivity, and coupled with sodium, fluorine, or organic material, Al is complexed as silicates, oxides, and hydroxides. Plants are unharmed by those molecules. However, as the pH of the soil decreases (pH < 5), Al changes into toxic Al3+, which is easily absorbed by plants [10]. 

The concentrations of Al increased over time in human plates [11]. It can be found in drinking water (as flocculant), industrially produced foods (as additive and packaging material), and fresh vegetables and fruits (available in soil) [12]. Moreover, utensils fabricated with Al and tea consumption are sources of Al in the human diet [8]. Since food is one of the main sources of Al [12], special attention should be paid to globally contaminated infant feeds and complementary foods [4,10,13].

In a recent published national study, the early introduction of infant formula and complementary feeding were common among Lebanese mothers, although exclusive breastfeeding rates were low [1]. Hence, the prevalence of bottle feeding between 0 and 6 months was 59.5%. In complementary feeding practices, only 47.1% of Lebanese mothers adhered to the WHO recommendations to introduce foods at 6 months [1]. The majority of children are fed infant formula from the first month because it is convenient, readily available, and, in certain circumstances, because the mother is unable to breastfeed due to a medical condition [14]. Breastfeeding or formula feeding should be supplemented with the age-appropriate safe feeding of nutritious solid foods starting at the age of 6 months. These foods can be prepared at home or purchased from the market, and the choice depends on the healthcare professional’s advice, the cognitive and physical status of infants, the mother’s employment status, and many social and economic conditions [11]. However, it is widely recognized that infant formulas and baby foods contain various concentrations of heavy metals. The raw materials used to produce baby and young child foods, such as milk, vegetables, fruit, and cereals, can be contaminated with Al, which poses a health threat in certain doses to children aged under five years [11]. Therefore, the aim of this study is to (1) assess the Al contamination level in infant formulas and baby foods available in the Lebanese market, and to (2) evaluate the pediatric health risk assessment through the calculation of exposure to aluminum due to the consumption of these foods among children aged under five years in Lebanon.

## 2. Materials and Methods

### 2.1. Sample Collection

The infant formula items (*n* = 41) and baby food products (*n* = 76) (biscuits: *n* = 7, cornflakes: *n* = 21, cereals: *n* = 16, and pureed foods: *n* = 32) were gathered from the Lebanese market, including pharmacies and grocery stores. The samples were collected on the basis of their availability in the Lebanese market. Prior to coding and storage in regulated humidity and temperature settings for analysis, all samples had had their expiration dates checked. Flame atomic absorption spectrometry (FAAS) was used to measure the Al content of each sample. Each food product’s means and standard deviations (SD) were calculated.

### 2.2. Determination of Aluminum Level

Infantile food items grouped into brands with different lot numbers were acquired in a number of three wrapped bundles. Mixed together, the food items were pulverized and homogenized. After that, 5 g of each sample was placed in a coded heat-resistant tube and dried at 100 °C in an oven to a constant weight. The dried samples were then weighed on a porcelain crucible (each marked with their code). Sequentially, the temperature was increased up to 500 °C within 1 h to dry-ash the samples in a muffle furnace. For an additional 12 h, dried samples were left to ash at this high temperature. After cooling, the obtained residue was dissolved in 1 M nitric acid and filtered using a Whatman filter paper into a flask with volume of 25 mL. Consequently, the solution was produced up to the line mark with 1 M nitric acid. The samples were coded, sealed, and stored in the laboratory refrigerator. Lastly, using a 309.3 nm Al lamp wavelength and a 0.7 nm slit width, the total Al content was ascertained with flame atomic absorption spectrometry (FAAS) (Shimadzu AA-6800 equipped with ACS 6100 auto sampler, Shimadzu, Tokyo, Japan). As an oxidant gas, nitrous oxide (N_2_O) was employed. Table 1 shows the optimized operating settings of the equipment as recommended by the manufacturer.

### 2.3. Validation

The method’s performance was determined using validation experiments. Information about limit of detection (LOD), limit of quantification (LOQ), uncertainty, recovery, precision, accuracy, and linearity are presented in Table 2.

A total of five different concentrations of Al standards were used to determine Al recoveries: 2, 5, 10, 15, and 20 mg/kg. The proportion of recovery ranged between 88% and 102%. A standard calibration curve that varied from 0.1 to 10 mg/kg was generated prior to sample analysis. Moreover, many linear regression equations were employed to determine the Al concentration in the tested samples. With a coefficient of determination of r2 > 0.9980, the calibration curve displayed good linearity. The standard deviation of the 12 blank measurements served as the basis for estimating the LOD and LOQ; LOD = 3 × SD and LOQ = 10 × LOD [15]. These values were 0.04 and 0.12 mg/kg, respectively [15]. The mean value of the certified reference material (CRM) method was used to calculate the accuracy, which included 0.4 mg/kg of Al, indicating high accuracy. CRM’s standard deviation of reproducibility was used to evaluate the precision. Our findings indicate that the examined samples had a satisfactory precision of 95.5%. The precision bias of CRM was combined to estimate the extended measurement of uncertainty, which was 11.8% (Table 2).

### 2.4. Exposure

Exposure was calculated for infants under five years old according to age, gender, and acceptable weight (kg) as reported by the Centers for Disease Control and Prevention (CDC) through the consumption of 41 infant formula samples and a total of 76 complementary food samples (16 cereals, 21 cornflakes, 7 biscuits, and 32 pureed foods) [16]. On the basis of the Al concentration in foods and infant consumption data, the estimated daily intake (EDI) of Al (mg/kg BW per day) was calculated using Equation (1) below [11]:

(1) $$\mathrm{EDI}\;(\mathrm{mg}/\mathrm{kg}\;\mathrm{BW}/\mathrm{day})=\frac{C\;\times \;\mathrm{Cv}\;}{\mathrm{BW}}$$

where Cv is the Al concentration, C is the daily intake from each sample, and BW is the body weight of infants.

### 2.5. Toxicological Contribution

The level of toxicological contribution (% of tolerable daily intake (TDI)) of the EDI can be calculated to the provisional tolerable weekly intake (PTWI) determined by the Joint FAO/WHO Expert Committee on Food Additives (JECFA) and the tolerable weekly intake (TWI) determined by European Food Safety Authority (EFSA) for Al according to Equation (2) below [11]:

(2) $$\%\;\mathrm{of}\;\mathrm{TDI}=\frac{\mathrm{Mean}\;\mathrm{EDI}\;}{\mathrm{PTDI}\;\mathrm{or}\;\mathrm{TDI}}\times 100$$

The PTWI for Al in foods according to the JECFA was used as a reference [17]. PTDI for Al is 286 μg/kg BW/day (PTWI  =  2 mg/kg BW/week) [18].

### 2.6. Hazard Quotient

The possible chronic health risk assessment index that is based on hazardous components was calculated as the hazard quotient (HQ). HQ ≥ 1 denotes a potential health concern, and indicates unacceptable food safety risk and that risk mitigation measures should be undertaken, whereas HQ < 1 shows no concern to health risk [19]. Equation (3) below was used to calculate HQ:

(3) $$\mathrm{HQ}=\frac{\mathrm{EDI}\;}{\mathrm{RfD}}$$

where RfD stands for oral reference dose (mg/kg/day). Since the US Environmental Protection Agency (US EPA) has not established a reference dose for Al, the recognized RfD for aluminum phosphide is 4 × 10^−4^ mg/kg/day based on a NOAEL of 0.51 mg/kg of food.

## 3. Results

### 3.1. Concentration of Aluminum in Infant Formula and Child Food Products

Al levels were analyzed in infant formulas, cereals, cornflakes, biscuits, and pureed foods using FAAS. The concentrations of Al in the samples, expressed as mean and ranges, are presented in Table 3. The lowest mean concentration of Al was in cereals (0.3 ± 5.7 × 10^−17^ mg/kg) compared to infant formulas (0.317 ± 0.038 mg/kg), cornflakes (0.361 ± 0.049 mg/kg), biscuits (0.357 ± 0.05 mg/kg), and pureed foods (0.362 ± 0.079 mg/kg) (Table 2). Overall, the mean contamination in 41 samples of infant formulas and 76 samples of baby foods was below the maximal permissible limit (MPL) required by FAO/WHO (400 μg/kg) [17] and the European Food Safety Authority (5000 to 10,000 μg/kg) [18].

### 3.2. Aluminum Exposure via Infant Formulas and Children’s Food Products

Aluminum’s risk assessment through the dietary exposure of infant formulas and complementary foods (biscuits, cereals, cornflakes, and pureed products) estimated the magnitude and the probability for harmful effects of Al on children under the age of five. As a cornerstone of the methodology for risk assessment, exposure assessment combines the quantities of aluminum in foods with consumption patterns, which yields useful data for risk management in the future. The recommended serving sizes and frequency of feedings for each infant age group were taken from the labels of the packages of infant formulas and complementary foods.

The EDI through infant formula intake in this study on average ranged between 0.0017 and 0.0081 mg/kg BW/day in males, and 0.0018 and 0.0087 mg/kg BW/day in females. Infants aged under 6 months faced the higher level of exposure to Al through the consumption of infant formulas, with an EDI ranging 0.0053–0.0081 mg/kg BW/day in males, and 0.0055–0.0087 mg/kg BW/day in females (Table 4). In addition, the EDI through pureed food intake was in the range of 0.005–0.015 mg/kg BW/day in males, and 0.006–0.016 mg/kg BW/day in females. The highest risk of exposure to Al in pureed food was among infants aged 8 to 10 months, with an EDI in the range of 0.0077–0.015 in males, and 0.0084–0.016 mg/kg BW/day in females (Table 4). Regarding cereal intake, the EDI was, on average, 0.0012 in males and 0.0013 mg/kg BW/day in females. The risk of exposure to Al in cereals was high in infants aged 6 to 8 months old, with an EDI of 0.0014 in males and 0.0016 mg/kg BW/day in females (Table 4). Regarding biscuit intake, the EDI was in the range of 0.00037–0.00068 in males and 0.0004–0.00075 mg/kg BW/day in females. The consumption of biscuits contaminated with Al posed the highest health risk among infants aged 6–8 months with an EDI in the range of 0.00051–0.00068 in males and 0.00057–0.00075 mg/kg BW/day in females (Table 4). The EDI through cornflake intake was in the range of 0.00071–0.0011 in males and 0.0007–0.0012 mg/kg BW/day in females. Through the consumption of cornflakes, infants aged 10–12 months are more exposed to Al, with an EDI range of 0.0008–0.001 in males and 0.0009–0.0012 mg/kg BW/day in females (Table 4).

### 3.3. Risk Assessment to Aluminum Exposure

Through the infant formula and baby food consumption, the average Al exposure among babies aged 0–23 months was 0.01 for males and 0.014 mg/kg BW/day for females. The highest Al exposure was found among infants aged 8–10 months. On the other hand, the lowest Al exposure was found in babies aged 3–5 months. Moreover, according to the JECFA regulations, the toxicological contribution of Al exposure to PTWI was < 6% for males and < 7% for females in all age categories (Table 5). The total Al exposure through infant formula and complementary food intake in both genders from all ages was below the tolerable weekly intake values set by JECFA (2 mg/kg of body weight per week) (Table 5). However, the HQ values exceeded 1 (Table 5).

## 4. Discussion

The current study found that total Al exposure through infant formula and complementary food intake at all ages in both genders was below the tolerable weekly intake values set by JECFA (2 mg/kg of BW per week), but HQ values exceeded 1. The highest Al level was detected in cornflakes (0.361 ± 0.049 mg/kg) and pureed foods (0.362 ± 0.079 mg/kg). The pediatric health risk assessment calculated through the EDI of Al was in the range of 0.0051–0.02058 mg/kg BW/day for males and 0.0055–0.02188 mg/kg BW/day for females.

To the best to our knowledge, our study is the first to investigate the pediatric health risk for exposure to Al in infant foods marketed in Lebanon. The concentration range of Al detected in infant formulas in the present study (0.3 to 0.4 mg/kg) was higher than the reported concentration in 2020 in Lebanon by Elaridi et al. (2020; 0.00008 to 0.00793 mg/kg) [4]. The increase in Al concentrations in these infant formula products in our study can be associated with the infant formula containers produced with Al. However, there is no research that conclusively shows that these packaged materials are a factor for the Al contamination of the powdered mixture.

### 4.1. Comparison with Other Arab Countries

There is a scarcity of data concerning the Al content in infant formulas and baby foods in the literature. In Saudi Arabia, Al concentration in infant formulas (1.6 to 1.9 mg/kg) is higher than that reported in our study (0.3 to 0.4 mg/kg) [20]. The same Saudi study reported the mean concentration of Al in cereals (9.88 ± 7.77 mg/kg), biscuits (5.83 ± 3.60 mg/kg), and pureed foods (6.45 ± 3.89 mg/kg) [20]; these concentrations were higher than the findings reported in our study.

### 4.2. Comparison with International Studies

In comparison to many other data concerning Al concentrations in infant formulas, our results are lower than those of Canada (0.018 to 1.10 mg/kg) [21], Brazil (0.14 to 5.94 mg/kg) [13], the United Kingdom (0.69 to 5.27 mg/kg) [22], and Turkey (0.7 to 6.987 mg/kg) [11]. The Al levels ascertained in this study conform to the maximal permissible limit for Al of 400 μg/kg determined by FAO/WHO in infant formulas [17]. Additionally, our findings show that Al levels in baby biscuits (0.3 to 0.4 mg/kg) were lower than those of Turkey (1.8 to 15.48 mg/kg) [11]. According to the European Food Safety Authority (EFSA) in 2008, high Al levels (5000 to 10,000 g/kg) were frequently found in breads, cakes, and pastries, with biscuits having the highest levels [23]. Further, the levels of Al in infant pureed foods varied from 0.2 to 0.4 mg/kg in the current study. The lowest amounts were found in samples from the second brand, including semolina, rice, carrots and turkey, zucchini with potatoes, fine sweet corn with mashed potatoes and turkey, fine vegetables and rice with veal, and carrots and potatoes with lamb (0.2 mg/kg). The highest levels (0.4 mg/kg) were in the first brand in all its samples. The levels of Al in pureed foods in our study were comparable to those reported in a study in France (0.189 to 0.653 mg/kg) [23], but lower than those in Ghana (2.89 to 11.07 mg/kg) [24] and Brazil (0.21 to 4.17 mg/kg) [25]. The levels of Al in infant cereals (0.3 mg/kg) in our study were lower than those reported in a study in Brazil (0.92 to 8.82 mg/kg) [25]. Moreover, our data show that the Al levels in cornflake samples were the highest (0.4 mg/kg), yet there is no study in the literature in which cornflake Al levels are described.

### 4.3. Risk Assessment of the Exposure of Lebanese Infants to Aluminum from Infant Formulas and Complementary Foods

Our results show a low EDI (<0.286 mg/kg BW/day) for all age groups. Referring to a previous national study published in 2020, our data show a lower mean EDI than that in their findings (EDI = 0.029 mg/kg BW/day) [4]. Our findings are similar to the results reported by a Brazilian study that showed a mean EDI of 0.01 mg/kg BW/day [13]. Further, our results were higher than those of a Turkish study published in 2022 that showed that the mean EDI was 0.00603 mg/kg BW/day [11]. Moreover, another Turkish study published in 2014 [26] and a British survey [27] showed higher EDI compared to our findings, with mean EDI values of 0.0335 and 0.1636 mg/kg BW/day, respectively. A French study also stated a higher EDI mean value (0.318 mg/kg BW/day) [28]. Our data also show lower mean EDI compared to that in a Nigerian study (EDI = 0.02 mg/kg BW/day) [2].

The calculated HQ for all age groups was > 1, which is consistent with our findings that Al exposure may be associated with a health risk. In the literature, there is a scarcity of studies in which HQ is investigated to assess possible health risk to Al exposure caused by the ingestion of baby foods. Only one Turkish study showed an HQ of 15, which is lower than the average HQ value in our study (25.5) [11].

On the other hand, the toxicological contribution of Al exposure was on average below 4%, which is similar to a recent Turkish study [11]. However, our findings are lower than those of Brazil (6.7%) [13], Nigeria (12.1%) [2], and Turkey (57.2%) [26]. The highest toxicological contribution in our study was among infants aged 8–10 months (5.72% and 6.11%, for males and females, respectively); this can be explained as follows: as babies grow, their intake of complementary foods increases from ¼ cup to a full cup, which leads to increased exposure to Al. Some studies suggest that the toxicological contribution to PTWI ranges from 37.9 to 66.8% [26].

Table 6 provides an overview of the few studies that investigated the exposure and the toxicological contribution of Al in infant formulas and baby food products.

### 4.4. Strength and Limitations

This study is the first of its kind in Lebanon to evaluate the Al concentration and the pediatric health risk assessment to Al in an infant population through the consumption of infant formulas and baby food products marketed in Lebanon. However, our study has the following limitations. The sample size is somewhat limited, as most of the brands represented by those samples are only offered in the Lebanese market. Additionally, due to the lack of Lebanese data, the information on baby weight used to calculate exposure was based on the CDC. Moreover, due to a dearth of research in this field in the Lebanese landscape, pediatric feeding data were derived from data on the labels of infant formulas and child food products rather than actual consumption. Lastly, the overall exposure to Al was not calculated on the basis of the consumption of other food categories that might also include Al. Therefore, future research should focus on collecting information about the baby food consumption and growth trends of Lebanese infants, using it to determine how much Al is being ingested by these infants through infant formulas and complementary foods.

## 5. Conclusions

Controlling Al contamination in the food chain is crucial because it has various negative consequences on health. Despite the fact that food contains high Al concentrations, our study showed lower exposure and toxicological contribution; however, a possible health risk is indicated. This shows that, despite the possible risk, no research has been performed to lower the amount of Al in baby foods. Hence, the best means of reducing dietary exposure include legislating on the matter, enforcing manufacturers to reduce the amount of Al in infant foods, and improving parents’ knowledge of these risks through training and development.

## Figures and Tables

**Table 1:** FAAS operating parameters for the determination of Al, Shimadzu cookbook.

Instrument Settings and Analytical Conditions of FAAS for the Determination of Al *				
Step Number	Temperature °C	Ramp Time, s	Heat	Internal N2 Flow L/min
1	60	3	RAMP	0.10
2	120	20	RAMP	0.10
3	250	10	RAMP	0.10
4	900	10	RAMP	1.00
5	900	10	STEP	1.00
6	900	3	STEP	0.00
7	2600	3	STEP	0.00
8	2600	2	STEP	1.00

* This table was adapted from the Shimadzu cookbook (Shimadzu AA-6800 equipped with ACS 6100 auto sampler, Shimadzu, Tokyo, Japan).

**Table 2:** Sensitivity, recovery, and precision indicators for Al in infant formulas and baby food products.

Type	Recovery (%)	Linearity	LOD (mg/kg)	LOQ (mg/kg)	Accuracy (%)	Precision (%)	Uncertainty (%)
Infant formulas and baby food products	88–102	0.9980	0.04	0.12	>97	95.5	11.8

LOD: limit of detection; LOQ: limit of quantification.

**Table 3:** Concentration of aluminum in infant powdered formula and in food products of children under the age of five.

Type	Number of Samples	Mean ± SD	Cv	MPL *
Infant formulas	41	0.317 ± 0.038 mg/kg	0.3–0.4 mg/kg	0.4 mg/kg
Cereals	16	0.3 ± 5.7 × 10^−17^ mg/kg	NA	5–10 mg/kg
Cornflakes	21	0.361 ± 0.049 mg/kg	0.3–0.4 mg/kg	5–10 mg/kg
Biscuits	7	0.357 ± 0.05 mg/kg	0.3–0.4 mg/kg	5–10 mg/kg
Pureed foods	32	0.362 ± 0.079 mg/kg	0.2–0.4 mg/kg	5–10 mg/kg

Cv: aluminum concentration. * Maximal acceptable limit, (MPL) for Al in infant formulas is 400 g/kg, as established by the FAO and WHO. Al levels in processed foods ranged from 5000 to 10,000 g/kg, according to the European Food Safety Authority; NA: not applicable.

**Table 4:** Exposure and pediatric health risk assessment to aluminum calculated through EDI and EWI among male and female infants aged under five through infant formula, pureed food, cereal, biscuit, and cornflake consumption at different ages.

Age	Infant Formula Intake	Cv	Average Body Weight (kg)		EDI (mg/kg BW/day)		EWI * (mg/kg BW/week)	
	Grams/day	mg/kg	Male	Female	Male	Female	Male	Female
0–1 weeks	77.4	0.3–0.4	3.8	3.7	0.0061–0.0081	0.0062–0.0083	0.043–0.057	0.044–0.058
1–4 weeks	86		4.3	4.5	0.006–0.008	0.0057–0.0075	0.042–0.056	0.040–0.053
2–8 weeks	107.5		5.3	5.2	0.006–0.0081	0.0061–0.0082	0.042–0.057	0.043–0.058
2–3 months	129		6.5	5.9	0.006–0.008	0.0066–0.0087	0.042–0.056	0.046–0.061
3–5 months	129		7.5	6.9	0.0051–0.0068	0.0055–0.0074	0.036–0.048	0.039–0.052
5–6 months	150.5		8.5	7.7	0.0053–0.007	0.0058–0.0078	0.037–0.049	0.041–0.055
6–8 months	120		9.3	8.4	0.0038–0.0051	0.0042–0.0057	0.027–0.036	0.03–0.04
8–10 months	90		10.2	9.3	0.0027–0.0036	0.0028–0.0038	0.019–0.025	0.02–0.027
10–12 months	60		10.9	10	0.0017–0.0021	0.0018–0.0024	0.012–0.015	0.013–0.017
	Pureed food consumption *							
6–8 months	262.5	0.2–0.4	9.3	8.4	0.005–0.011	0.006–0.012	0.039–0.079	0.043–0.087
8–10 months	393.75		10.2	9.3	0.0077–0.015	0.0084–0.016	0.054–0.108	0.059–0.118
10–12 months	393.75		10.9	10	0.0071–0.014	0.0078–0.015	0.05–0.101	0.055–0.110
12–23 months	393.75		12.5	11.7	0.0062–0.012	0.0067–0.013	0.044–0.088	0.047–0.094
	Cereal consumption *							
6–8 months	44	0.3	9.3	8.4	0.0014	0.0016	0.0099	0.011
8–10 months	44		10.2	9.3	0.0013	0.0014	0.009	0.0099
10–12 months	44		10.9	10	0.0012	0.0013	0.0085	0.0092
12–23 months	44		12.5	11.7	0.00104	0.00114	0.0073	0.0078
	Biscuit consumption *							
6–8 months	16	0.3–0.4	9.3	8.4	0.00051–0.00068	0.00057–0.00075	0.0036–0.0048	0.004–0.0053
8–10 months	16		10.2	9.3	0.00045–0.00061	0.00051–0.00068	0.0032–0.0043	0.0036–0.0048
10–12 months	16		10.9	10	0.00042–0.00058	0.00047–0.00062	0.003–0.0041	0.0033–0.0044
12–23 months	16		12.5	11.7	0.00037–0.0005	0.0004–0.00054	0.0026–0.0035	0.0028–0.0038
	Cornflake consumption *							
10–12 months	30	0.3–0.4	10.9	10	0.0008–0.0011	0.0009–0.0012	0.0057–0.0077	0.0063–0.0084
12–23 months	30		12.5	11.7	0.00071–0.00095	0.0007–0.001	0.0050–0.0067	0.0053–0.0071

Cv: aluminum concentration; EDI: estimated daily intake; TDI: tolerable daily intake; EWI: estimated weekly intake (https://www.cdc.gov/growthcharts/html_charts/wtageinf.htm, accessed on 30 March 2022). The calculation was based on the 75th percentile. * We estimated the average weekly intake (EWI) by multiplying the previously ascertained EDI by 7; the consumption patterns of infant pureed food were derived from the “practical guidance on the quality, frequency, and amount of food” to offer to children aged 6–23 months; the consumption patterns of cereals were derived from the “guidelines on the amount of cereals” per day of 6–12 months infants detailed on the cereal package labels; the consumption patterns of biscuits was calculated according to one 8 g biscuit as mentioned on the product labels; the consumption patterns of cornflakes were calculated according to one 30 g portion of cornflakes as mentioned on the product labels, and noninfant cereals are recommended to be introduced after 9 months of age.

**Table 5:** Total exposure and pediatric health risk assessment to aluminum among male and female infants aged under five years through infant formula and complementary food consumption at different ages, calculated through EDI, the % of TDI, HQ, and EWI.

Total Exposure	Range EDI (mg/kg/BW/day)		Mean EDI (mg/kg/BW/day)		% of TDI		HQ		EWI * (mg/kg BW/day)	
Age	Male	Female	Male	Female	Male	Female	Male	Female	Male	Female
0–1 weeks	0.0061–0.0081	0.0062–0.0083	0.0071	0.00725	2.48	2.53	17.7	18.1	0.043–0.057	0.044–0.058
1–4 weeks	0.006–0.008	0.0057–0.0075	0.007	0.0066	2.44	2.3	17.5	16.5	0.042–0.056	0.040–0.053
2–8 weeks	0.006–0.0081	0.0061–0.0082	0.00705	0.00715	2.46	2.5	17.6	17.8	0.042–0.057	0.043–0.058
2–3 months	0.006–0.008	0.0066–0.0087	0.007	0.00765	2.44	2.67	17.5	16.1	0.042–0.056	0.046–0.061
3–5 months	0.0051–0.0068	0.0055–0.0074	0.00595	0.00645	2.08	2.25	14.9	16.1	0.036–0.048	0.039–0.052
5–6 months	0.0053–0.007	0.0058–0.0078	0.0088	0.0068	3.07	2.37	22	17	0.037–0.049	0.041–0.055
6–8 months	0.01071–0.01818	0.01237–0.02005	0.014445	0.01621	5.05	5.66	36.1	40.5	0.0795–0.1297	0.088–0.1433
8–10 months	0.01215–0.02058	0.01311–0.02188	0.016365	0.017495	5.72	6.11	40.9	43.7	0.0852–0.1463	0.0925–0.1597
10–12 months	0.01122–0.01898	0.01227–0.02052	0.0151	0.016395	5.28	5.73	37.7	40.9	0.0792–0.1363	0.0868–0.149
12–23 months	0.00832–0.01449	0.00919–0.01568	0.011405	0.012435	3.98	4.34	25.8	31.1	0.0589–0.1055	0.0629–0.1127
Average	0.0051–0.02058	0.0055–0.02188	0.01	0.0104	3.49	3.6	25	26	0.069	0.07

EDI: estimated daily intake; TDI: tolerable daily intake; HQ: hazard quotient; EWI: estimated weekly intake. * We estimated the average weekly intake (EWI) by multiplying the previously ascertained EDI by 74.

**Table 6:** Exposure assessment for aluminum in infant and child food samples from different countries around the world.

Country	Year	EDI (mg/kg BW/day)	% TDI	References
Lebanon	2022	0.01	3.5	Current study
Turkey	2022	0.00603	2.11	[11]
Lebanon	2020	0.029	NA	[4]
Nigeria	2020	0.02	12.1	[2]
Brazil	2019	0.01	6.7	[13]
France	2018	0.318	NA	[28]
Turkey	2014	0.0335	57.2	[26]
United Kingdom	2006	0.1636	NA	[27]

EDI: estimated daily exposure; TDI: tolerable daily intake.

## Data Availability

The data of the present study are available from the corresponding author upon request.
